# Acute cytomegalovirus proctitis and epididymitis acquired via sexual transmission in an immunocompetent patient: a case report

**DOI:** 10.1186/s13256-023-04216-1

**Published:** 2023-11-10

**Authors:** Deborah M. Oyeyemi, Elizabeth Chan, Mason Montano, Annika Belzer, Onyema Ogbuagu, Heidi Zapata, Jessica J. Tuan

**Affiliations:** 1 Department of Internal Medicine, Yale University School of Medicine, New Haven, CT USA; 2 Section of Infectious Disease, Yale University School of Medicine, 135 College St., Suite 323, New Haven, CT 06510 USA

**Keywords:** Cytomegalovirus, Proctitis, Epididymitis, Sexually transmitted infections

## Abstract

**Background:**

We present a case report of an immunocompetent host with presumed sexually transmitted cytomegalovirus proctitis and epididymitis, where there currently is a sparsity of published data.

**Case presentation:**

A 21-year-old previously healthy Caucasian individual was admitted for severe rectal and testicular pain in the setting of proctitis and epididymitis. Serology and rectal pathology confirmed acute primary cytomegalovirus infection.

**Conclusions:**

This report details his diagnostic workup and highlights cytomegalovirus as a rare cause of sexually transmitted disease among immunocompetent persons.

## Background

Cytomegalovirus (CMV) is a herpes virus that infects individuals of all ages. CMV is transmitted through blood, tissue, and bodily fluids, including saliva, urine, and semen. Infected immunocompetent persons are often asymptomatic but can develop a mononucleosis-like syndrome, characterized by systemic symptoms including fever and lymphadenopathy, and sore throat [1]. Immunocompromised patients, including transplant recipients, those on chronic immunosuppressant therapy, and individuals with advanced human immunodeficiency virus (HIV), are at increased risk of developing serious primary and secondary CMV infections that require medical attention; these include retinitis, colitis, and esophagitis [2]. Older adults and immunocompromised hosts are at increased risk of CMV reactivation secondary to immune dysregulation—impaired cell-mediated immunity—that occurs with aging [3].

Primary CMV infections are extremely rare among young, healthy adults [1, 4]. Here we report a case of CMV proctitis and epididymitis in a young immunocompetent patient.

## Case presentation

A 21-year-old Caucasian patient assigned male at birth presented with 2 days of severe rectal, abdominal, testicular pain, and loose stools. He reported history of hemorrhoids with intermittent bright red blood per rectum over 9 months. History was notable for prior appendectomy and allergies to penicillin (rash), pollen, and several raw fruits/vegetables. He took emtricitabine/tenofovir/disoproxil fumarate for HIV pre-exposure prophylaxis. He recently received prophylactic chlamydia treatment and one dose of the mpox vaccine series approximately 1 month prior to presentation. He was a student. He had a non-monogamous relationship with a male partner and reported receptive and insertive anal intercourse with inconsistent condom use and intermittent use of sex toys, including anal beads. He had recently traveled to Hawaii, California, Michigan, and Maine in the summer. Two years prior to presentation he had traveled to Japan. He had multiple pet dogs and cats. He reported having a younger sibling with lymphomatoid papulosis.

In the emergency room, vital signs were normal. On examination, he had tender bilateral inguinal lymphadenopathy, mild right lateral testicular tenderness when palpated, and tenderness in the right lower abdominal quadrant and suprapubic region. No hemorrhoids or anal fissures were visualized. Initial workup included complete blood count remarkable for low platelet count of 125,000 cells/µL (reference: 150,000–420,000 cells/µL) and increased atypical lymphocytes at 3% (reference: 0–1%); white blood cell count was normal at 6000 cells/µL (reference: 3800–10,800 cells/µL). Computed tomography (CT) of the abdomen/pelvis showed mesorectal lymphadenopathy and rectal wall thickening with circumferential fat stranding consistent with acute proctitis, without abnormalities of the prostate, testicle, or epididymis. Basic metabolic panel, urinalysis, and throat/urine/rectal gonorrhea/chlamydia testing were normal. He received ketorolac for pain and was started on ceftriaxone/metronidazole/doxycycline prior to medical admission for further management of proctitis.

He was promptly switched from ceftriaxone to ciprofloxacin after a scrotal/testicular ultrasound (performed 12 hours after the CT abdomen) confirmed left epididymitis. He was transitioned from ceftriaxone to ciprofloxacin to cover for *Pseudomonas*, which is a common bacterial organism that can be associated with epididymitis. On hospitalization day 2, metronidazole and doxycycline were discontinued, and acyclovir was started for empiric herpes simplex virus (HSV) coverage. Additional infectious workup was notable for detection of CMV on initial quantitative polymerase chain reaction (PCR) testing of the blood, albeit with quantitation < 137 IU/mL (reference: < 137 IU/mL). CMV immunoglobulin M (IgM) was negative, as were the following studies: Monospot testing, hepatitis panel (including hepatitis C antibody), treponemal antibody, rectal mpox/HSV/varicella zoster virus (VZV) PCRs, repeat gonorrhea/chlamydia (urine/rectal/throat), respiratory viral panel, serum HIV PCR, serum enterovirus and adenovirus PCRs, tick-borne serology panel, and urine mycoplasma/ureaplasma PCR. He had stool studies performed including *Helicobacter pylori* stool antigen, *Clostridium difficile* enzyme immunoassay, stool ova and parasites, stool pathogen PCR panel (*Campylobacter*, *Salmonella*, *Shigella*, *Yersinia enterocolitica*, Shiga Toxin 1 and 2, *Vibrio*), and stool Giardia antigen that were negative. Calprotectin had initially been sent due to concern for possible inflammatory bowel disease and was noted to be elevated to 1110 µg/g (reference: < 50 µg/g).

**Fig. 1:**
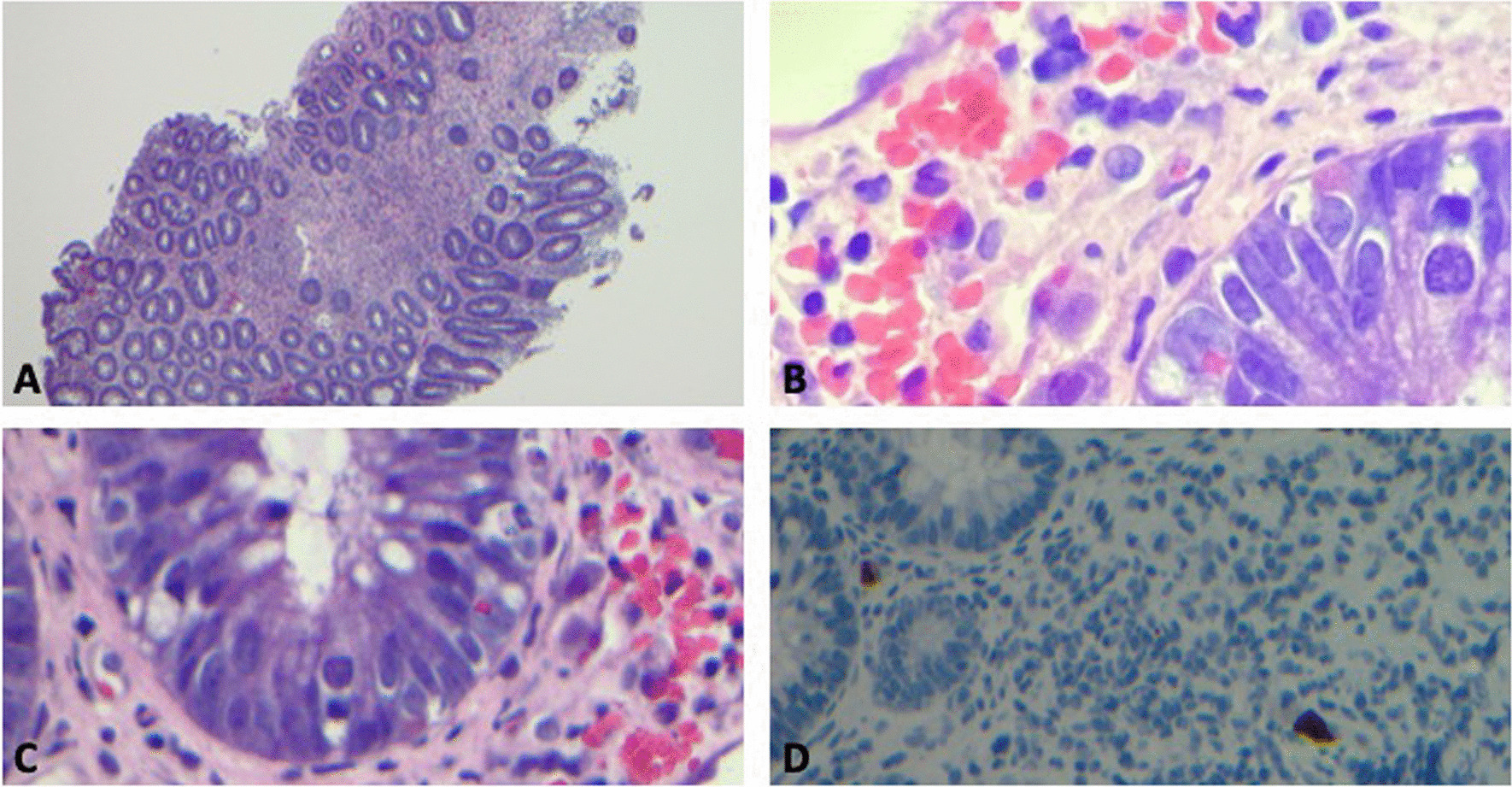
Histopathologic examination of the rectal biopsy sample of the patient with proctitis and cytomegalovirus (CMV) infection. **A** Rectal biopsy sample shows ulcerated mucosa with increased apoptotic bodies. **B**, **C** Rectal biopsy sample shows enlarged atypical cells with viral inclusions. “Owl eye” inclusion bodies are visualized. **D** Cytomegalovirus-positive immunostaining from rectal biopsy sample is demonstrated

Over a period of days, he also developed myalgias and arthralgias. Due to lack of improvement in rectal pain over a period of days, colorectal surgery and gastroenterology advised additional workup with colonoscopy. On hospital day 7, colonoscopy was remarkable for irregular ulcerations with exudate in the distal rectum, mild-to-moderate patchy erythema in the more proximal rectum, and mild mucosal edema in the sigmoid colon. Biopsies were taken from the terminal ileum, colon, and rectum and sent for tissue culture and pathology. Rectal pathology demonstrated findings consistent with CMV-associated proctitis (Fig. 1). These findings included patchy inflammatory changes in the rectum with ulceration, ischemic-type changes, and prominent apoptosis within crypts. CMV inclusions were seen in the stroma and focal epithelial cells, and had positive CMV immunostaining. Colonic and ileal mucosa samples had no significant abnormalities and did not show findings of inflammatory bowel disease. Immunohistochemistry stains for *Treponema* spp. and HSV were negative.

Valganciclovir was initiated for treatment of CMV proctitis; all other antibiotic/antiviral treatments were discontinued. Repeat Epstein–Barr virus (EBV)/CMV quantitative viral load testing and serologies were sent. The EBV serum panel result was consistent with prior infection. The initial CMV IgG was mildly positive at 0.28 U/mL (reference: < 0.20 U/mL). Add-on studies from hospital day 4 showed that CMV IgG had been negative (reference: < 0.20 U/mL), while CMV IgM had become positive at 70.60 AU/mL (reference: < 8 AU/mL) (Table 1). On hospital day 10, CMV IgG and IgM were both positive at 1.4 U/mL (reference: < 0.20 U/mL) and > 240 AU/mL (reference: < 8 AU/mL), respectively. Given the evolution in serology with rising CMV IgM titers and development of positive IgG over time, the clinical presentation was thought to be consistent with acute primary CMV infection. A T lymphocyte subset panel was also sent to determine CD4 count and evaluate for possible undiagnosed immunodeficiency. He had a low-normal CD4 count of 513 cells/µL (reference: 490–1740 cells/µL), elevated CD8 count of 1867 cells/µL (reference: 153–980 cells/µL), and a low CD4/CD8 ratio at 0.27 (reference: 0.73–5.86). A decreased CD4/CD8 ratio can be seen with viral infections, including CMV infection.

**Table 1:** Cytomegalovirus serology and polymerase chain reaction testing results during hospitalization

	Day 1	Day 4	Day 10
CMV IgM (AU/mL)	(−) < 8 [< 8]	(+) 70.6 [< 8]	(+) > 240 [< 8]
CMV IgG (U/mL)	(+) 0.28 [< 0.20]	(−) < 0.20 [< 0.20]	(+) 1.40 [< 0.20]
CMV PCR (IU/mL)		(+) < 137 [< 137]	(+) 1430 [< 137]
CMV quantitation (log_10_) (log IU/mL)		(−) < 2.14 [Linear range: 137–9,100,000 IU/mL]	(+) 3.16 [Linear range: 137–9,100,000 IU/mL]

*CMV* cytomegalovirus, *PCR* polymerase chain reaction, *IgG* immunoglobulin G, *IgM* immunoglobulin M

The hospital course was also complicated by mildly elevated liver transaminases that trended upward to peak of 189 U/L (reference: 10–35 U/L), prior to discharge on day 14. CT of the abdomen and pelvis with intravenous contrast had not shown any hepatobiliary pathology, and a viral hepatitis panel was negative. The transaminitis was ultimately attributed to CMV infection.

The patient was discharged with a 3-week course of valganciclovir and recommended to follow-up with primary care, infectious diseases, and immunology physicians. He was counseled to abstain from sexual intercourse until treatment completion. At a follow-up visit with the infectious diseases physician 3 weeks later, the patient had significantly improved with near complete abatement of rectal and testicular pain, myalgias, and arthralgias. Repeat liver function tests 2 weeks post-hospital discharge were normal.

## Discussion and conclusions

This report features a rare case of acute primary CMV proctitis and epididymitis in an immunocompetent host. It is likely that the patient acquired the infection via sexual transmission, either from possible anoreceptive intercourse with his sexual partner and/or use of the aforementioned anal beads. Though repeat gonorrhea and chlamydia tests were negative, he did receive empiric treatment with ceftriaxone and doxycycline in the case of false-negative testing, in addition to receiving valganciclovir for CMV infection. In recent years, a few case reports have been published regarding CMV proctitis in immunocompetent patients [5, 6]. In fact, one report suggested that CMV proctitis is an underdiagnosed sexually transmitted illness (STI), particularly among men who have sex with men (MSM) and HIV-infected patients [5].

CMV proctitis is typically described as a secondary reactivation, which may present asymptomatically or with mild symptoms in immunocompetent individuals [7]. Additionally, proctitis is a less common manifestation of CMV when compared with other gastrointestinal tract illnesses or even retinitis. While one review from 1980 highlighted the condition among MSM, most of the patients included were immunocompromised [8]. Additional research is needed to evaluate the prevalence of CMV proctitis among non-HIV-infected immunocompetent patients.

While this patient was treated with valganciclovir for 3 weeks, there is a lack of consensus on antiviral therapy duration for CMV proctitis in the young, healthy population. National guidelines for the management of sexually transmitted proctitis comment on considering the clinical syndrome of CMV as a diagnosis in the setting in HIV-infected immunosuppressed patients. Clinical guidelines may need to adapt their diagnostic frameworks, if this condition is found to be more prevalent among healthy individuals [9].

One consideration during this case was whether our seemingly healthy patient had an underlying immunodeficiency, particularly after he was found to have T-cell abnormalities. This patient had a low CD4/CD8 ratio with normal-low CD4 and elevated CD8 counts, which may have been driven by CMV. He followed up with an immunologistpost-discharge and had a repeat lymphocyte subset panel that showed normal CD4 and CD8 T cell counts. He was recommended to have additional immune testing outpatient. Studies have shown that CMV infection is associated with CD8 T cell expansion, which may explain the normalization of our patient’s T-cell lymphocyte counts following treatment [10]. He continued to follow up with immunology and infectious disease physicians outpatient.

In conclusion, we report a rare case of CMV proctitis and epididymitis, which was probably sexually transmitted, in an immunocompetent patient. We recommend that clinicians consider CMV on their differential diagnosis for proctitis in both immunocompetent and immunocompromised patients, particularly if they engage in anoreceptive intercourse and other sex practices like use of beads or sharing sex toys. More research is needed to evaluate the prevalence of this condition among healthy patients to better inform current guidelines for STI management.

## Data Availability

The data that support the findings of this study are available but protected under the institutional review board at Yale given the sensitive nature of patient health information, and thus, restrictions apply to the availability of these data, which were used under license for the current study, and so are not publicly available. Data are, however, available from the authors upon reasonable request and with permission of Yale.
